# *Blind Insight*: Metacognitive Discrimination Despite Chance Task Performance

**DOI:** 10.1177/0956797614553944

**Published:** 2014-12

**Authors:** Ryan B. Scott, Zoltan Dienes, Adam B. Barrett, Daniel Bor, Anil K. Seth

**Affiliations:** 1 School of Psychology; 2 Sackler Centre for Consciousness Science; 3 School of Informatics, University of Sussex

**Keywords:** insight, judgment, consciousness, decision making, cognitive neuroscience, open data, open materials

## Abstract

Blindsight and other examples of unconscious knowledge and perception demonstrate dissociations between judgment accuracy and metacognition: Studies reveal that participants’ judgment accuracy can be above chance while their confidence ratings fail to discriminate right from wrong answers. Here, we demonstrated the opposite dissociation: a reliable relationship between confidence and judgment accuracy (demonstrating metacognition) despite judgment accuracy being no better than chance. We evaluated the judgments of 450 participants who completed an AGL task. For each trial, participants decided whether a stimulus conformed to a given set of rules and rated their confidence in that judgment. We identified participants who performed at chance on the discrimination task, utilizing a subset of their responses, and then assessed the accuracy and the confidence-accuracy relationship of their remaining responses. Analyses revealed above-chance metacognition among participants who did not exhibit decision accuracy. This important new phenomenon, which we term *blind insight*, poses critical challenges to prevailing models of metacognition grounded in signal detection theory.

The phenomenon of blindsight (Weiskrantz, Warrington, Sanders, & Marshall, 1974) has had a powerful influence on the development of psychology and neuroscience because it challenges the intuition that metacognitive awareness must necessarily accompany discriminative accuracy. Studies of blindsight, which may be exhibited following damage to the primary visual cortex, demonstrate that substantial decision accuracy (e.g., discriminating between visual stimuli) can occur in the absence of metacognitive insight into that ability; blindsight patients classically report being blind to the stimuli that they so deftly categorize. In this article, we introduce a related phenomenon that has the potential to similarly transform psychology’s understanding of metacognition and its relationship to the distinction between conscious and unconscious processing. We term this phenomenon *blind insight*, and it can be thought of as the reverse of blindsight; it is characterized by accurate metacognitive discrimination (i.e., knowing when you are right or wrong) in the reliable absence of decision accuracy.

Metacognition, and in particular the ability to assess the accuracy of knowledge states, is fundamental to understanding executive processes (e.g., Koriat, 2007), the nature of memory (e.g., Mazzoni, Scoboria, & Harvey, 2010), good educational practice (e.g., Koriat, 2012), gambling (Lueddeke & Higham, 2011), development (Beck, McColgan, Robinson, & Rowley, 2011), cognitive differences between species (Smith, Beran, Couchman, Coutinho, & Boomer, 2009), social interaction (Frith, 2012), mental illness (Hamm et al., 2012), and the distinction between conscious and unconscious processes in both perception (Kanai, Walsh, & Tseng, 2010) and learning (Dienes & Seth, 2010).

Given the importance of metacognition to such a wide variety of research endeavors, there has been a strong motivation both to refine its accurate bias-free measurement and elucidate the underlying cognitive architecture. Signal detection theory (SDT) provides a useful method to measure stimulus-discrimination accuracy independently of response bias (Lau, 2008; Lau & Passingham, 2006; Macmillan & Creelman, 2005) and has been widely adopted and extended for the assessment of metacognition (Barrett, Dienes, & Seth, 2013; Galvin, Podd, Drga, & Whitmore, 2003; Ko & Lau, 2012; Maniscalco & Lau, 2012; Rounis, Maniscalco, Rothwell, Passingham, & Lau, 2010; see Fig. 1). The measure of sensitivity provided by SDT is generally termed Type I *d*′ (*d*′_1_) when computed for stimulus discrimination and Type II *d*′ (*d*′_2_) when computed for metacognitive discrimination. For a given two-alternative forced-choice judgment, *d*′_1_ provides an estimate of the relationship between response and target, and *d*′_2_ provides an estimate of the relationship between confidence and accuracy. In addition to providing a mechanism for measuring metacognition, the SDT framework has formed the basis of a variety of theoretical models of metacognition, decision making, and awareness more generally (Clifford, Arabzadeh, & Harris, 2008; Maniscalco & Lau, 2012; Pleskac & Busemeyer, 2010; Scott & Dienes, 2008; Snodgrass, Bernat, & Shevrin, 2004). However, while the benefit of and rationale for applying SDT to measure metacognition is clear, its adoption in cognitive models of metacognition is less well justified.

**Fig. 1.**
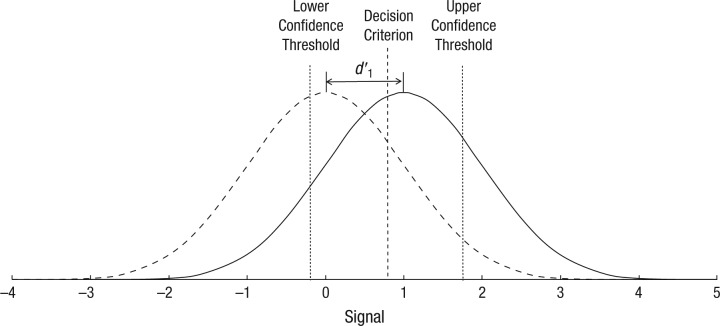
Schematic illustrating general principles of signal detection theory. The dashed curve shows the signal distribution when the stimulus is absent (or ungrammatical, new, etc.), and the solid curve shows the distribution when the stimulus is present (or grammatical, old, etc.). The index *d* ′_1_ is the distance between the means of these two distributions in units of the standard deviation of the stimulus-absent distribution. The stimulus is classified as present or absent depending on whether the signal is, respectively, greater or less than the (Type I) decision criterion. There is confidence in that judgment if the signal is greater than the upper confidence threshold or lower than the lower confidence threshold.

In its classical form, SDT offers a hierarchical framework whereby information available to the metacognitive judgment derives from the same signal exploited by the first-order discriminative process. Indeed, it can be theoretically demonstrated that an SDT framework (with some straightforward assumptions) cannot give rise to metacognitive insight in the absence of decision accuracy (Barrett et al., 2013). While there is intuitive appeal of an arrangement in which confidence in a judgment derives from the strength of the signal driving the first-order decision, a purely bottom-up hierarchical configuration is at odds with both neuroanatomical and neurophysiological evidence. A growing body of data indicates that both bottom-up (feed-forward) and top-down (feedback, recurrent) connections and processing make crucial contributions to perception, with the latter being particularly vital to attentional grouping and awareness (Bowman, Schlaghecken, & Eimer, 2006; Jaskowski & Verleger, 2007; Salin & Bullier, 1995).

Research exploring individual differences in metacognition is similarly suggestive of interactions between low-level sensory decisions and metacognitive processes. Fleming, Weil, Nagy, Dolan, and Rees (2010) demonstrated that individual differences in metacognitive performance on a perceptual decision task were correlated with gray-matter volume in the anterior prefrontal cortex and white-matter microstructure connected with this region. Crucially, the anterior prefrontal cortex receives input from higher-order cortical regions rather than from early sensory regions, which is consistent with a role in metacognitive judgment rather than in simple perceptual decisions. In contrast, other decision-making-related regions (e.g., posterior parietal cortex) receive inputs from early sensory regions and have been shown to support the primary perceptual decision (Kiani & Shadlen, 2009). Other researchers have demonstrated a dissociation between reaction times and confidence that is also at odds with typical models of how confidence arises (Wilimzig, Tsuchiya, Fahle, Einhaeuser, & Koch, 2008).

Although the application of SDT to metacognition enjoys increasing popularity, it is by no means the only approach to modeling the confidence-accuracy relationship. The metamemory literature offers a range of theoretical approaches based on concepts such as cue utilization (Koriat, 2007). In cue utilization, factors as diverse as fluency and brightness have been shown to influence confidence (Busey, Tunnicliff, Loftus, & Loftus, 2000; Oppenheimer, 2008), though such cues can be unrelated to the accuracy of first-order judgments and, therefore, may not provide a veridical source of metacognition.

In the research reported here, we focused specifically on the SDT framework. We sought to evaluate whether metacognition and first-order decision accuracy can be dissociated in a manner incompatible with the SDT framework and, in so doing, offer clear constraints on the type of model able to account for this characteristically human cognitive process. To accomplish this, we examined metacognitive performance in artificial-grammar learning (AGL; Pothos, 2007; Reber, 1967), a paradigm in which after incidental exposure to apparently random strings of letters, participants classify new strings as obeying or contravening an inherent set of rules. The AGL task has proven particularly useful in the study of implicit learning and is well known for demonstrating decision accuracy in the absence of confidence (i.e., a knowledge state equivalent to blindsight; e.g., Dienes, Altmann, Kwan, & Goode, 1995). Here, we revealed the opposite dissociation—blind insight—by establishing an unbiased selection of participants who exhibited chance performance and then examining their metacognitive accuracy.

## Method

### Participants

Participants were 450 student volunteers (227 male, 223 female) ages 18 to 40 years (*M =* 22, *SD =* 3.53), each of whom were paid £3 or given course credit to take part in one of eight AGL experiments. The current study used all data available from standard AGL studies completed in the first author’s research lab at the University of Sussex in the previous 5 years.^1^ All participants were naive to the experimental hypotheses and were randomly assigned to condition in each study. All experiments received ethical approval by the University of Sussex Life Sciences ethics committee and were conducted in accordance with the Declaration of Helsinki.

### Materials

Two finite-state grammars (Grammar A and Grammar B, both from Reber, 1969) were used to generate grammar strings between five and nine characters in length. Training sets comprised either 15 or 16 strings (depending on the experiment) selected from the grammar to which the participant had been assigned and repeated three times in random orders. The test set comprised either 60 or 64 strings (depending on the experiment), including half from each grammar that had not been used during training. Strings were presented in black on a white background at the center of a computer screen.

### Procedure

Training strings were presented under the guise of a short-term memory task, with each string presented for memorization for 5 s, followed by a brief recall task before the next string appeared. The presentation order of both training and test strings was separately randomized for each participant. After training, participants were informed that the order of letters in the training strings had obeyed a complex set of rules and that they were to classify a new set of strings, exactly half of which would obey the same rules. Test strings were presented one at a time, and participants were asked to indicate the following without time constraints: (a) how familiar the string seemed to them on a scale from 0 to 100, (b) whether or not the string was grammatical (i.e., obeyed the rules), (c) how confident they were in their grammaticality judgment on a scale from 50 to 100 (50 *=* 50:50 chance of being right or wrong, 100 = complete certainty of being right), and (d) the basis for their grammaticality judgment (random guessing, intuition, familiarity, a rule or rules they had derived, or recollection).

### Design

A dual-grammar design was employed in which half the participants were trained on Grammar A and half on Grammar B. At test, all participants classified the same set of test strings, all of which were different from the training strings. Precisely half of the test strings conformed to Grammar A, and half conformed to Grammar B. Thus, the nongrammatical test strings for one group were grammatical for the other group, which eliminated the need for an untrained control group. The key independent variable was grammatical status, manipulated within subject (grammatical vs. ungrammatical). There were two dependent variables of interest: first-order decision accuracy, for which we computed *d*′ for the relationship between grammaticality judgments and the true grammatical status (*d*′_1_), and metacognition, for which we computed *d*′ for the relationship between confidence (no confidence whatsoever vs. some degree of confidence) and accuracy, typically termed Type II *d*′ (*d*′_2_). An alpha of .05 was adopted for all significance tests.

## Results

### Approach to analysis

Our objective was to assess whether reliable metacognitive accuracy could exist in the absence of first-order accuracy. To test this, we identified that subset of participants whose decision accuracy was equivalent to chance. To avoid incorrect inferences, it was important that this selection be robust to biases arising from regression toward the mean. Specifically, analysis needed to be conducted on a sample of trials that had not itself been subject to the bias imposed by the selection process. This was accomplished by selecting participants on the basis of a subset of their trials and analyzing the remainder.

**Fig. 2.**
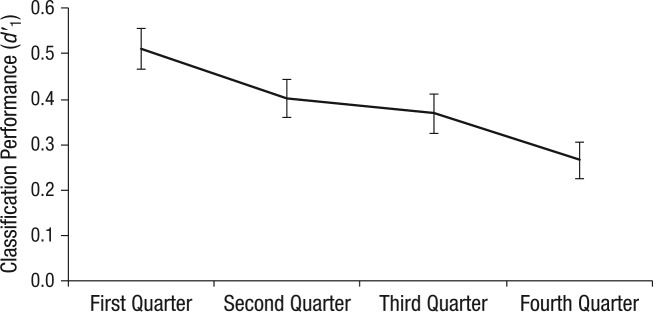
Mean classification performance (*d* ′_1_) for each quarter of the test phase. Error bars indicate ±*1 SEM. n* = 283.

While a repeated random subsampling method might typically be applied to select trial subsets in a maximally unbiased fashion, our data contained a predictable linear trend that precluded this approach. It is an established phenomenon in AGL that performance (*d*′_1_) declines across the test phase of a dual-grammar design (e.g., see Mealor & Dienes, 2013, who demonstrated this for the same dual-grammar design used in the experiments analyzed here). A likely explanation for the effect is the increasing interference that ungrammatical strings—encountered only during the test phase—have on the representation of valid grammatical strings. This same linear trend over time was significant in the present data, *F*(1, 282) = 17.43, *p* < .001 (see Fig. 2).

Where performance changes systematically in this way, chance performance on a random subsample of trials cannot reliably predict chance performance on the remainder. Consequently, we adopted a linear sampling approach (i.e., selecting participants who performed at chance in early test trials and analyzing their later trials), thus taking advantage of the tendency for performance to decrease over time. For this approach to be effective, the selected subset needs to be sufficiently large that performance for that subset is representative of performance across subsequent trials. We first attempted a selection including participants for whom *d*′_1_ was less than or equal to 0 for the first 50% of trials. However, 50% did not provide a sufficiently representative estimate of *d*′_1_; the *d*′_1_ for the remaining trials was significantly greater than zero (*M* = 0.20, *SE* = 0.08), *t*(52) = 2.57, *p* = .013, *d* = 0.36, which illustrates the issue of regression toward the mean and prevented the intended analysis. We therefore selected participants for whom *d*′_1_ was less than or equal to 0 for the first 75% of trials. This percentage provided a sufficiently representative estimate such that *d*′_1_ for these participants was not significantly different from zero in the remaining trials (*M* = −0.06, *SE* = 0.11), *t*(32) = −0.49, *p* = .626, *d* = 0.09.

The same selection process was applied to identify participants who reliably performed above chance (*d*′_1_ > 0) so as to permit metacognitive performance to be contrasted in those who did and did not exhibit decision accuracy. Only participants for whom both *d*′_1_ and *d*′_2_ could be computed for the analysis subset (i.e., who had nonzero counts in every cell) were included; above chance: *n* = 165, at chance: *n* = 33. The number of participants reliably performing at chance was, as anticipated, relatively small. However, simulations conducted with these numbers (reported later) provide reassurance that our findings did not arise from sample-size bias.

We further computed a Bayes factor to establish the extent to which these data provide evidence for the null hypothesis (*d*′_1_ = 0) for the at-chance group, rather than simply reflecting insensitivity. Adopting the procedure advocated by Dienes (2011), the alternative hypothesis was *d*′_1_ following a half-normal distribution with a standard deviation equivalent to the *d*′_1_ observed for participants showing above-chance decision accuracy in the first 75% of trials (0.38). The resulting Bayes factor of 0.19 was less than one-third and hence represents strong evidence for the null hypothesis. This selection process thus provided an unbiased sample of participants who did not exhibit first-order accuracy and, therefore, permitted us to examine their metacognitive performance under these circumstances.

### Metacognitive accuracy in the absence of first-order accuracy

**Fig. 3.**
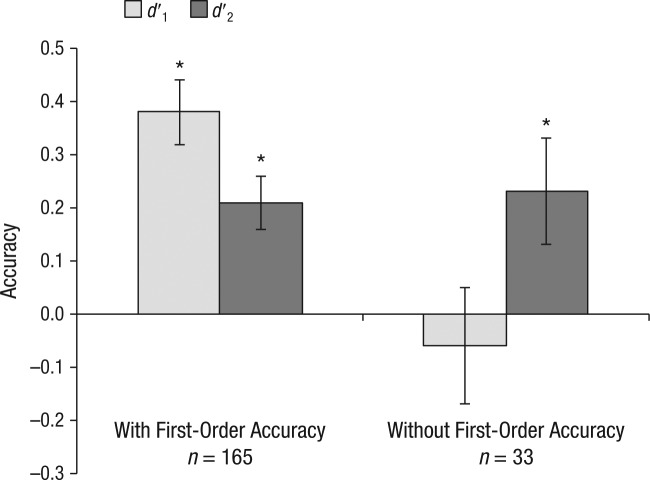
Mean first-order accuracy (*d* ′_1_) and metacognitive accuracy (*d* ′_2_) for the analysis trials, separately for participants who did and did not exhibit above-chance first-order accuracy in the selection trials. Error bars indicate ±1 *SEM*. Asterisks indicate results significantly different from zero (*p* < .05).

Figure 3 illustrates the mean *d*′_1_ and *d*′_2_ for the analysis trials (final 25%); results are plotted separately for participants who performed above chance in the selection trials (first 75%) and those who performed at or below chance in the selection trials. Analyses were conducted listwise to ensure that the means for each index were based on the same participants. Among participants who exhibited first-order accuracy in the selection trials, *d*′_2_ was significantly greater than chance, *t*(164) = 4.65, *p* < .001, *d* = 0.36, and showed the typical relationship with *d*′_1_, specifically that the mean of *d*′_2_ is approximately half that of *d*′_1_ (Krueger, Klapoetke, & Mattler, 2011). Crucially, among participants who did not exhibit first-order accuracy, *d*′_2_ remained significantly greater than chance, *t*(32) = 2.30, *p* = .028, *d* = 0.40, and was not significantly different from the *d*′_2_ of participants who did exhibit first-order accuracy, *t*(196) = 0.17, *p* = .868, *d =* 0.03. Thus, the analysis revealed reliable metacognitive performance among participants who did not exhibit first-order decision accuracy.

Although these analyses were based on *d*′_2_, alternative measures of metacognition revealed the same pattern of results. Meta-*d*′, devised by Maniscalco and Lau (2012) to be independent of bias due to the Type I criterion, was significantly above chance among participants who did not exhibit first-order accuracy (*M* = 1.10, *SE* = 0.43), *t*(32) = 2.57, *p* = .015, *d* = 0.45, and nonsignificantly different from that observed among participants who exhibited first-order accuracy (*M* = 0.72, *SE* = 0.17), *t*(196) = 0.87, *p* = .384, *d* = 0.16.

Similarly, the difference in the percentage of correct judgments when participants were confident versus not confident, known as the *confidence-accuracy slope* (Dienes & Seth, 2010), was again significantly greater than zero among participants who did not exhibit first-order accuracy (mean difference = 9%, *SE* = 4%), *t*(32) = 2.26, *p* = .031, *d* = 0.42, and nonsignificantly different from the slope observed among participants who exhibited first-order accuracy (mean difference = 8%, *SE* = 2%), *t*(196) = 0.021, *p* = .836, *d* = 0.05. Regardless of the index used, the results reflect the same underlying phenomenon: Participants’ confidence reports expressed reliable knowledge of when their grammaticality judgments were right or wrong despite those same grammaticality judgments failing to discriminate grammatical from ungrammatical strings.

### The source of metacognitive accuracy

**Fig. 4.**
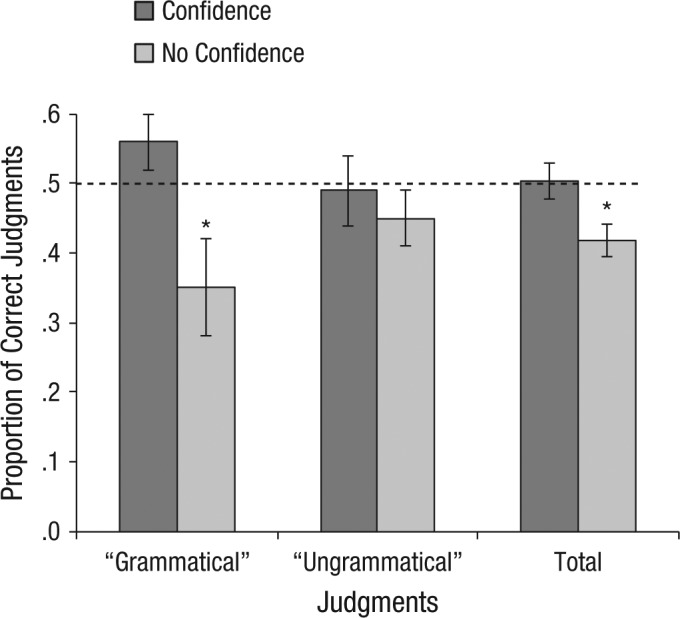
Mean proportion of correct grammaticality judgments among participants who did not exhibit first-order accuracy. Results are shown for “grammatical,” “ungrammatical,” and all judgments made with and without confidence. Error bars indicate ±1 *SEM*. The dashed line indicates chance performance. Asterisks indicate results significantly different from chance (*p* < .05). *n* = 33.

To explore the source of the observed metacognition seen in participants lacking decision accuracy, we conducted a 2 (judgment: grammatical vs. ungrammatical) × 2 (confidence: confident vs. guess) within-subject analysis of variance on the proportion of correct judgments (see Fig. 4). This analysis revealed no main effect of judgment, *F*(1, 26) = 0.15, *p* = .702, η_*p*_^2^ = .01; a marginal effect of confidence, *F*(1, 26) = 3.91, *p* = .059, η_*p*_^2^ = .13; and a marginal judgment-by-confidence interaction, *F*(1, 26) = 3.12, *p* = .089, η_*p*_^2^ = .11. These findings reflect the fact that the proportion of correct judgments was below chance for guesses, *t*(32) = 3.36, *p* = .002, *d* = 0.58, but close to chance for judgments made with some confidence, *t*(32) = 0.14, *p* = .890, *d* = 0.02, and that this difference was marginally stronger for grammatical than for ungrammatical classifications (cf. Fleming & Dolan, 2010; Higham, Perfect, & Bruno, 2009).

Overall, these findings suggest that the observed metacognition (in the absence of decision accuracy) reflects a tendency for judgments made without confidence to exhibit below-chance accuracy, while confident judgments remain at chance. One possible interpretation of this difference is that there was some form of implicit error monitoring taking place that was expressed as reduced confidence where a wrong answer was made. If this was the case, however, the information exploited by the error-monitoring process was clearly unavailable to the preceding classification judgment.

### Effect of delay between judgments

Participants made grammaticality and confidence judgments consecutively, with confidence judgments necessarily following the grammaticality judgments. This arrangement gives rise to two potential issues. The first is that during the momentary pause between judgments, participants’ knowledge state may continue to stabilize, and metacognitive performance, deriving from the latter judgment, may show greater accuracy as a result. Some evidence for this has been identified in the context of reaction-time responses (Baranski & Petrusic, 2001; Charles, Van Opstal, Marti, & Dehaene, 2013; Pleskac & Busemeyer, 2010). However, in our experiments, there was no time constraint on judgments and, therefore, no obvious reason why a (first-order) judgment would be made before a stable knowledge state had been achieved. Furthermore, when Tunney and Shanks (2003, Experiments 1a and 1b) contrasted *d*′_2_ for confidence judgments made simultaneously with grammaticality judgments with those made following the grammaticality judgment (as we did here), they observed a nonsignificant reduction in *d*′_2_ (mean difference = −0.10, *SE* = 0.18), *t*(22) = 0.54, *p* = .593, *d* = 0.77, a finding that is more consistent with metacognition decaying during the delay rather than increasing. The *d*′_2_ observed in the current study (*M* = 0.23, *SE* = 0.10) is also significantly larger than the change they observed, *t*(54) = 1.70, *p* = .048 (one tailed), which provides further reassurance that the effect was not the result of the sequential arrangement of judgments.

A second issue arises from the potential for participants to make errors when reporting grammaticality judgments. As there were no time constraints, very few errors were anticipated; nonetheless, if a participant were confident that a string was grammatical but inadvertently pressed “ungrammatical,” or vice versa, then they might choose to report no confidence to reflect that error. Assuming they were applying veridical knowledge, this could result in below-chance accuracy for judgments attributed no confidence. For example, if we assume that participants’ knowledge on average permitted 60% classification accuracy (10% above chance), then when applied without error, their judgments would have 60% accuracy and be reported to have been made with some confidence. In contrast, when they applied that knowledge but inadvertently pressed the wrong button (and realized this), the judgments would have 40% accuracy (10% below chance) and would be reported to have been made with no confidence. As can be seen, the maximum extent to which the accuracy of no-confidence judgments could be reduced below chance by this mechanism is limited to the equivalent above-chance accuracy of confident judgments (10% in this illustration). Therefore, if this account applies, we should have observed above-chance accuracy in confident judgments at least equivalent to the below-chance accuracy of judgments made without confidence. This was not observed. Judgments without confidence were 8% below chance (*M* = 42%, *SE* = 2%), so the accuracy of confident judgments would need to be at least 58% (8% above chance). In fact, confident judgments were numerically at chance (*M* = 50%, *SE* = 2%), significantly less accurate than 8% above chance (*M* = 58%, *SE* = 2%), *t*(32) = 2.27, *p* = .030, *d* = 0.39. Thus, the absence of confidence in incorrect judgments cannot have arisen from inadvertently pressing the wrong button.

### Inequality of variances

In an SDT model, if the underlying signals exploited to classify grammatical and ungrammatical strings had unequal variances, this could in principle result in an inflated estimate of *d*′_2_ (Barrett et al., 2013). While it is impossible to observe any putative grammaticality signal directly, previous research has shown that subjective ratings of familiarity for test strings strongly predict both grammaticality judgments and confidence ratings (Scott & Dienes, 2008, 2010a). We therefore used the variance in these subjective familiarity ratings to estimate that of the underlying grammaticality signals. While the ratio of variances for familiarity ratings attributed to grammatical versus ungrammatical strings did not differ significantly from 1 (*M* = 1.29, *SE* = 0.19), *t*(25) = 1.60, *p* = .122, *d* = 0.30, we nonetheless undertook a simulation analysis to establish the likelihood of the observed *d*′_2_ resulting from this ratio.

The parameters detailed here apply to both the following simulation and the criterion-jitter simulation described in the following section: On each trial, the grammaticality signal was generated as a Gaussian random variable, with *M* = 0 and *SD* = 1 for an ungrammatical string, and *M* = *d*′_1_ and *SD* = 1.27 for a grammatical string (i.e., the ratio of variances was set at 1.62, the upper end of the 95% confidence interval, to make it most likely that our simulations could explain away the results); the decision criterion was based on the observed proportion of strings judged to be grammatical (*M* = 0.47, *SE* = 0.03); the confidence thresholds were based on the observed proportions of guesses for grammatical (*M* = 0.32, *SE* = 0.05) and ungrammatical classifications (*M* = 0.52, *SE* = 0.04); and each simulated experiment included the same number of participants and trials on which the empirical analysis was conducted.

We simulated the experiment 1,000 times, assuming a *d*′_1_ of zero. The observed probability of obtaining a value of *d*′_2_ as large as the empirically observed value of 0.23 was *p* = .010. Thus, we can conclude that the observed metacognition is unlikely to represent an artifact resulting from inequality of variances.

### Criterion jitter

If the criterion employed in making grammaticality judgments was subject to jitter, this could result in an underestimate of *d*′_1_ while potentially leaving *d*′_2_ relatively unchanged (Mueller & Weidemann, 2008), again potentially accounting for our main findings. We therefore conducted a second SDT simulation to evaluate the extent to which the estimated *d*′_1_ of zero may have resulted from criterion jitter. Given the most commonly observed ratio between *d*′_1_ and *d*′_2_ of 2:1 (Kunimoto, Miller, & Pashler, 2001), to obtain a *d*′_2_ approximately equal to the observed 0.23, we assumed the true *d*′_1_ was equal to 0.46. We then implemented a substantial jitter in the criterion by giving it a Gaussian distribution of standard deviation 1.5. Note that this level of jitter is the logical maximum assuming that the signal distributions overlap by approximately 50%, and the criterion is based solely on the last observed string (instead of a more stable running average). Simulating the experiment 1,000 times with these parameters revealed that the probability of observing *d*′_1_ as low as zero was *p* = .002. The mean simulated estimate of *d*′_1_ was 0.26 (*SE* = 0.003), which is substantially greater than the upper bound of the 95% confidence interval on the empirically observed value (*M* = *−*0.06, 95% confidence interval = [−0.29, 0.18]). Thus, we can conclude that the presence of substantial metacognition observed among participants who did not exhibit first-order accuracy (*d*′_1_ = 0) was not due to an underestimate of *d*′_1_ resulting from criterion jitter.

## Discussion

We exploited the AGL paradigm to evaluate metacognitive performance in participants who lacked first-order decision accuracy. Analysis was conducted on data independent of that used in the selection of participants, and additional analyses and simulations eliminated effects of a delay between judgments, unequal variance, and criterion jitter as alternative explanations for the findings. The results revealed significant metacognitive discrimination independent of first-order decision accuracy. Specifically, confidence reports expressed reliable knowledge of whether judgments had been right or wrong despite the judgments themselves showing chance levels of discrimination. While the phenomenon of blindsight challenges the intuition that metacognitive performance must necessarily follow from reliable decision accuracy, the phenomenon of blind insight challenges the intuition that decision accuracy must necessarily exist for there to be metacognitive discrimination of the veracity of those first-order judgments. While we see no reason to expect this phenomenon to be unique to the context of AGL, additional research is needed to determine the extent to which our results generalize across distinct paradigms, including perceptual decisions.

What are the implications of our results for theoretical models of metacognition? Models that rest on SDT fit naturally with bottom-up hierarchical arrangements in which low-level discriminations provide the signals supporting high-level metacognitive discriminations. These models can naturally account for dissociations between (low-level) decision accuracy and metacognition as seen in blindsight and unconscious knowledge by simply assuming that a failure in the metacognitive process can leave lower-level discriminative processes intact. In contrast, blind insight represents a dissociation that is fundamentally at odds with a purely bottom-up hierarchical relationship relating first-order decision processes to metacognition, because the absence of reliable decision accuracy precludes the availability of signals supporting above-chance metacognitive performance on these models. Our observation of blind insight therefore establishes that the metacognitive process must either draw on information additional to that available to the first-order decision process or exploit the same information in a substantially different way. Such an arrangement is not readily implemented by models that adhere closely to SDT in assuming that metacognitive judgments are made on the same signal underlying first-order decisions (Clifford et al., 2008; Maniscalco & Lau, 2012; Pleskac & Busemeyer, 2010; Scott & Dienes, 2008; Snodgrass et al., 2004). While amendments to these models might accommodate the blind-insight phenomenon, any such amendments would represent a fundamental departure from the standard SDT framework. In short, significant metacognition (*d*′_2_ > 0) in the absence of first-order accuracy (*d*′_1_ = 0) is incompatible with a classical signal detection framework (see Barrett et al., 2013).

To account for blind insight therefore requires a model architecture that less closely couples metacognitive performance to the signal driving first-order judgments. Progress in this direction has been made by Timmermans, Schilbach, Pasquali, and Cleeremans (2012), who describe a “hybrid” neural network model in which first-order decision processes and second-order metacognitive processes are supported by independent networks. While both networks are feed-forward architectures trained using standard back-propagation algorithms, the metacognitive network takes as input not simply the output of the first-order network but rather the difference between its input and output. It is interesting that during training on a blindsight simulation, this model exhibited a pattern of results similar to blind insight; however, this was only a transient stage of model dynamics rather than a stable state as in our data. Moreover, their model remains faithful to the assumptions of SDT by proposing unidirectional bottom-up signal flow (back-propagation is used only for updating connection strengths).

Given the inability of SDT-based models to account for blind insight, our data suggest that a more radical revision of metacognition models is required. One potential direction for revision would take into account the evidence, mentioned in the Introduction, that neural dynamics underlying perceptual decisions involve counterflowing bottom-up and top-down neural signals (Bowman et al., 2006; Jaskowski & Verleger, 2007; Salin & Bullier, 1995). A framework for interpreting these countercurrent dynamics is provided by *predictive processing*, which proposes that top-down projections convey predictions (expectations) about the causes of sensory signals, with bottom-up projections communicating mismatches (prediction errors) between expected and observed signals across hierarchical levels, with their mutual dynamics unfolding according to the principles of Bayesian inference (Clark, 2013). Future models of metacognition could leverage this framework to propose that both first-order and metacognitive discriminations emerge from the interaction of top-down expectations and bottom-up prediction errors, for example by allowing top-down signals to reshape the probability distributions of evidence on which decision thresholds are imposed (Barrett et al., 2013). We can at this stage only speculate as to whether such a model might provide the means to account for the blind-insight phenomenon and recognize that predictive coding is just one among a variety of potential frameworks that could be applied to that challenge (Timmermans et al., 2012).

In summary, blind insight demonstrates a previously undescribed dissociation between second-order awareness and first-order performance and in so doing presents a critical challenge to prevailing models of metacognition.

## Supplementary Material

Supplementary material

## Notes

1. Data for the present study were drawn from all participants in the four experiments reported in Scott and Dienes (2008; *N* = 80 each), Experiment 1 of Scott and Dienes (2010b; *N* = 40), and three unpublished studies (*N* = 30 each). Sample sizes for all of the experiments were based on estimated numbers required to achieve reasonable power for the individual objectives of each experiment. In each case, data collection stopped when that predetermined number was met.
