# Supplementary material for: Blind Insight: Metacognitive Discrimination Despite Chance Task Performance
Source: Psychol Sci. 2014 Dec;25(12):2199–208. doi: 10.1177/0956797614553944 (PMC4263819; doi:10.1177/0956797614553944)
Supplement: Supplementary material [file DS_10.11770956797614553944.pdf]

OPEN PRACTICES DISCLOSURE

PLEASE COMPLETE AND RETURN TO EDITORIALOFFICE@PSYCHOLOGICALSCIENCE.ORG

***Psychological Science* manuscript #: PSCI-13-0874.R2** **Corresponding author: Ryan Scott**

Articles accepted to *Psychological Science* after January 1, 2014, are eligible to earn badges that recognize open scientific practices: publicly available data, material, or preregistered research plans. Please read more about the badges here (https://osf.io/tvyxz/wiki/view/) and in the FAQs here (https://osf.io/tvyxz/wiki/faq/).

☐ **Please check this box if you are not interested in participating.**

To apply for one or more badges acknowledging open practices, please provide the information requested in the relevant sections below. **Your responses will be posted with your article as supplemental online material.**

You will not qualify for a badge for a given item unless you can provide a URL, doi, or other permanent path for accessing the specified information in a public, open-access repository AND answer "yes" to the relevant question #2. (Please see additional qualifiers in the section on preregistration.) If you answer "no" to question #2, but would still like your article to include a URL/doi/etc. to data/materials/registered research plans that are publicly available, we will publish the information in a note, but your article will not receive the associated badge.

Qualifying public, open-access repositories are committed to preserving data, materials, and/or registered analysis plans and keeping them publicly accessible via the web into perpetuity. Examples include the Open Science Framework (OSF; https://osf.io/) and the various Dataverse networks. Hundreds of other qualifying data/materials repositories are listed at http://re3data.org/ and http://databib.org/. Preregistration of an analysis plan must take place via a publicly accessible registry system (e.g., OSF, ClinicalTrials.gov or other trial registries in the WHO Registry Network, institutional registration systems). **Personal websites and most departmental websites do not qualify as repositories.**

There are, of course, circumstances in which it is not possible or advisable to share data, materials, or a research plan publicly. For example, there are cases in which sharing participant data could violate confidentiality. If you would like your article to include a note with an explanation of such circumstances, please provide the explanation in the Alternative Note section below.

Open Data:

1. Provide the URL, doi, or other permanent path for accessing the data in a public, open-access repository:

   https://osf.io/ivdk4/files/

2. Is there sufficient information for an independent researcher to reproduce the reported results? If no, explain.

   Yes.

Open Materials:

1. Provide the URL, doi, or other permanent path for accessing the materials in a public, open-access repository:

   https://osf.io/ivdk4/files/

2. Is there sufficient information for an independent researcher to reproduce the reported methodology? If no, explain.

   Yes

Preregistration:

1. Provide the URL, doi, or other permanent path to the registration in a public, open-access repository.*

2. Was the analysis plan registered prior to examination of the data or observing the outcomes? If no, explain.**

3. Were there additional registrations for the study other than the one reported? If yes, provide links and explain.*

4. Were there any changes to the preregistered analysis plan for the primary confirmatory analysis? If yes, explain.**

5. Are all of the analyses described in the registered plan reported in the article? If no, explain.*

*No badge will be awarded if (1) is not provided, **or** if (3) is answered “yes” without strong justification, **or** if (5) is answered “no” without strong justification.

**If the answer to (2) is “yes,” the notation DE (Data Exist) will be added to the badge, indicating that registration postdates realization of the outcomes but predates analysis. If the answer to (4) is “yes” with strong justification for changes, the notation TC (Transparent Changes) will be added to the badge, indicating that the analysis plan was altered but the preregistered analyses and rationale for the change are provided.

Alternative Note:
